# DTAP: a unified graph transformer framework for joint prediction of drug–target affinity and docking pose

**DOI:** 10.1093/bib/bbag069

**Published:** 2026-02-16

**Authors:** Junxi Liu, Yulian Ding, Yan Yan, Liangzhen Zheng, Yi Pan

**Affiliations:** Southern University of Science and Technology, Shenzhen 518055, China; Computer Science and Artificial Intelligence, Shenzhen University of Advanced Technology, Shenzhen 518107, China; Nanjing Hanwei Public Health Research Institute Co., Ltd, Nanjing 210000, China; Central for High Performance Computing, Shenzhen Institute of Advanced Technology, Chinese Academy of Sciences, Shenzhen 518055, China; Nanjing Hanwei Public Health Research Institute Co., Ltd, Nanjing 210000, China; Shenzhen Zelixir Biotech Company Ltd., Shenzhen 518107, China; Computer Science and Artificial Intelligence, Shenzhen University of Advanced Technology, Shenzhen 518107, China; Shenzhen Key Laboratory of Intelligent Bioinformatics, Shenzhen Institute of Advanced Technology, Chinese Academy of Sciences, Shenzhen 518055, China

**Keywords:** drug–target affinity, docking poses, pretrained large model, graph transformer, multi-task learning

## Abstract

Predicting drug–target interactions (DTIs) is crucial for modern drug discovery. However, existing machine learning models have significant limitations: they are typically designed for a single task—either predicting binding affinity or docking pose—leading to excellent performance on one metric but limited practical utility. These models also often struggle with generalizability to novel molecules and proteins due to their reliance on small, labeled datasets. Furthermore, they frequently ignore the essential information contained within the 3D structure of proteins and molecules. To overcome these challenges, we introduce DTAP, a unified framework that simultaneously predicts both the quality of docking poses and drug–target binding affinity. To boost its generalizability, DTAP leverages pretrained large models to learn rich, contextual representations of drugs and targets from extensive unlabeled data. The framework also directly incorporates 3D structural data from both molecules and proteins, using two graph transformers to learn their joint representations. A shared latent vector and task-specific decoders enable crucial cross-task knowledge transfer, allowing the model to learn from the interconnected nature of these two properties. DTAP significantly outperforms state-of-the-art methods on both tasks, demonstrating superior performance especially in cold start situations where data are scarce. Our interpretability analysis on the model’s attention mechanisms confirms its ability to effectively focus on key binding sites. All results indicate that DTAP is a valuable and practical tool for accurately predicting drug–target affinities and docking poses.

## Introduction

Traditional drug development is characterized by high costs, lengthy timelines, and extremely low success rates. On average, developing a new drug requires $\sim $$2.6 billion and 10–15 years, yet only $\sim $0.1% of candidate compounds progress to clinical trials, with merely 12% ultimately gaining regulatory approval [1–3]. This inefficiency primarily stems from the vast drug-like molecular search space, which encompasses $\sim 10^{23}-10^{60}$ possible structures, while the number of therapeutically valuable molecules is estimated at only $10^{8}$. To tackle this, computational methods have become essential for predicting drug–target interactions (DTI) and accelerating the initial discovery phase.

A critical step in computational drug discovery is predicting drug–target affinity (DTA) that quantifies the strength of a drug’s binding to its target. Binding affinity can be quantified through experimentally derived metrics such as inhibition constant (Ki), dissociation constant (Kd), and half-maximal inhibitory concentration (IC50). These parameters enable the calculation of binding energy between drugs and targets, facilitating subsequent drug screening and prioritization. Another crucial task is binding pose prediction that involves determining the precise 3D orientation of a small molecule within a protein’s active site [4, 5]. Accurate pose prediction is vital for rational drug design, allowing researchers to optimize a molecule’s pharmacological properties [6].

Historically, these tasks relied on experimental techniques like X-ray crystallography. However, these methods often suffered from low throughput and limited accuracy. To overcome these challenges, computational methods employing binding energy scoring functions were developed. Early scoring rules from the 1980s, however, relied on simple polynomials and were insufficient for accurately describing the complex interactions of drug–target complexes [7]. The subsequent rise of machine learning and deep learning has revolutionized this field. Initial models, like DGDTA [8] and BiComp-DTA [9], used long short-term memory (LSTM) networks to predict binding affinity [10]. Although effective, these sequential models could not effectively incorporate molecular spatial information. To address this, more advanced models like GraphDTA [11] and MGraphDTA [12] adopted Graph Neural Networks (GNNs) [13] that are better suited for processing the graph-structured data of molecules [14, 15]. More recently, models such as AttentionDTA [16] and AttentionMGT-DTA [17] have integrated attention mechanisms to improve both prediction accuracy and model interpretability by focusing on key docking regions.

A significant challenge for deep learning models is their dependence on large-scale, high-quality labeled training datasets. However, the currently available data resources often suffer from insufficient labeled data, consequently limiting the generalization capability of trained models. When confronted with complex or novel molecular structures, these models often struggle to achieve reliable predictions. This is similar to the cold start problem in recommendation systems. With the advancement of pretrained large model (LM) technologies, some researchers have begun to integrate pretrained molecular and protein embeddings from LMs that provide a foundation of highly generalized features. For instance, models like AttentionMGT-DTA [17] leverage pretrained protein LM embeddings to enhance DTA prediction performance. DTIAM employs self-supervised pretraining on extensive label-free datasets to learn robust representations of drugs and targets. These pretrained representations significantly enhance the model’s performance in various downstream prediction tasks, including DTI, DTA, and drug mechanism prediction [18]. T-GraphDTA predicts DTA by protein pretraining model and hybrid graph neural network [19]. By incorporating these pretrained LM representations, those methods not only improve their prediction accuracy but also demonstrate better adaptability to diverse and previously unseen molecular structures.

Despite these advancements in scoring power, recent studies reveal a significant practical limitation: machine learning models for DTA prediction, when trained solely on native structures, often underperform in real-world docking and virtual screening scenarios [20]. This highlights a crucial challenge, as effective drug discovery requires not only predicting binding strength but also accurately identifying the correct binding orientation (pose) and efficiently screening for active compounds. To enhance real-world applicability, researchers have developed various predictors aimed at identifying near-native ligand poses or improving active compound screening. For instance, DeepBSP [21] directly incorporates the root-mean-square deviation (RMSD) between predicted and native poses as an additional criterion, prioritizing spatial structural similarity. DeepDock [22] utilizes a graph neural network to learn the distance probability distribution between protein–ligand atoms, moving beyond direct affinity or RMSD prediction. Meanwhile, DeepRMSD+Vina [23] integrates modified traditional force field terms (van der Waals and Coulombic) as features, proving effective in enhancing docking power and pose optimization. However, a persistent issue across these sophisticated methods is their inconsistent performance across multiple evaluation metrics simultaneously. Achieving high accuracy in both binding affinity and precise pose prediction, alongside robust virtual screening, remains a complex hurdle. This has led to a growing consensus among researchers on the critical importance of balancing performance across docking and screening tasks. Furthermore, few of these deep learning methods offer directly interpretable indicators with physical meanings that are essential for intuitive guidance in computational drug development [21, 24]. The challenge of accurately predicting optimal ligand binding poses and DTA continues to largely persist, underscoring the ongoing need for more integrated predictive frameworks.

In summary, existing machine learning models are typically single-task, designed to predict either binding affinity or docking pose, which often leads to strong performance in one area but limited practical utility in a real-world setting. These models also struggle with generalizability to new molecules and proteins, a problem known as a “cold start,” because they rely on small, labeled datasets for training. Furthermore, existing approaches usually neglect the crucial information contained within the 3D structure of proteins and molecules. To overcome those limitations, we developed DTAP, a unified framework capable of simultaneously predicting molecular–protein binding affinity and scoring the RMSD between docking poses and native structures. To leverage crucial structural information and focus on native conformations, DTAP applies two Graph-Transformer encoders to process the 3D structures of molecules and proteins, respectively. Furthermore, to overcome the cold start problem and boost the model’s generalizability on unseen compounds, DTAP incorporates powerful representations from pretrained LMs that capture rich substructural and contextual details from extensive unlabeled data. Most importantly, to address the challenge of balancing virtual screening and docking performance, DTAP is designed as a multitask framework. The binding affinity and docking pose prediction tasks share a common latent vector, which enables effective knowledge transfer between them, while using task-specific decoders to generate the final predictions. This integrated design significantly enhances the framework’s practical utility. DTAP demonstrates superior performance on standard DTA datasets (Davis and KIBA [25, 26]) and exhibits strong generalization capabilities across various cold-start scenarios. Furthermore, it achieves a high Top-1 docking success rate on the CASF-2016 benchmark [27] and shows strong correlations in binding affinity prediction and ranking. Through interpretability analysis, we show the model’s ability to focus on critical binding sites. In summary, our work presents a highly accurate and versatile molecular–protein interaction prediction model that could significantly accelerate the drug discovery process.

## Materials and methods

### Datasets

**Figure 1:**
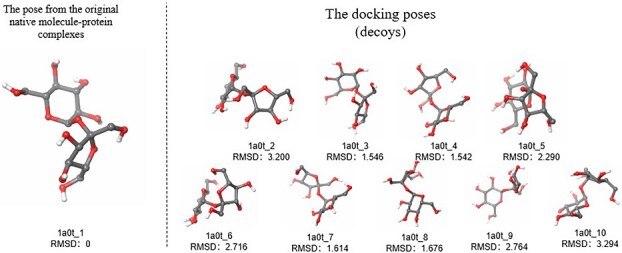
Structural variations in docking poses for identical molecules.

In this study, we employed the PDBbind database along with the Davis and KIBA datasets for training purposes. PDBbind dataset is used for both affinity prediction task and the docking pose prediction task, as it includes the affinity value and docking poses of native molecule–protein complexes. To enhance the diversity of ligand binding conformations, we performed re-docking of the original native molecule–protein complexes using AutoDock Vina [28]. The molecules and proteins were preprocessed with tools such as PyMOL, and docking boxes were defined accordingly. Batch docking was then carried out using Vina. Following docking, an average of 10 new docked conformations (decoys) were generated for each native molecule–protein complex, and the corresponding root-mean-square deviation (RMSD) values relative to each native complex were calculated. One of the native molecule–protein complexes and its corresponding decoys are illustrated in Fig. 1. After removing data that could neither be processed by the software nor by the pretrained models included in our framework, as well as data that could potentially cause data leakage, we retained high-quality 124 784 complexes as our training set. The CASF-2016 dataset, after similar filtering of data, served as the corresponding independent test set of PDBbind.

The Davis and KIBA datasets are applied to test the performance of protein–ligand binding affinity prediction. Binding affinity is a key measure of how strongly a molecule, like a drug inhibitor, binds to a protein. This is typically expressed using values like the Kd, Ki, and IC50. In the Davis dataset, binding affinity is determined using the Kd value. This value quantifies the specific dissociation measurements between a kinase protein and its associated inhibitor. For the KIBA dataset, a specific method called KIBA was used. This method combines the statistical information from Kd, Ki, and IC50 values to ensure consistency. The affinity values in this dataset primarily range from 10 to 13, with a concentration around 11. To improve the quality of the data for model training, duplicate entries were removed from both datasets. Table 1 shows the summary statistics of the refined benchmark datasets.

**Table 1:** Summary of the benchmark datasets

**Dataset**	**Molecule**	**Protein**	**Complex**	**Active**	**Inactive**
Davis	68	361	24 616	1649	22 967
KIBA	2111	228	117 948	24 543	93 405
PDBbind	124 784 (Different poses)	2641	124 784	15 455	109 329
CASF2016	11 334 (Different poses)	54	11 334	2598	8736

### Overview of the DTAP framework

**Figure 2:**
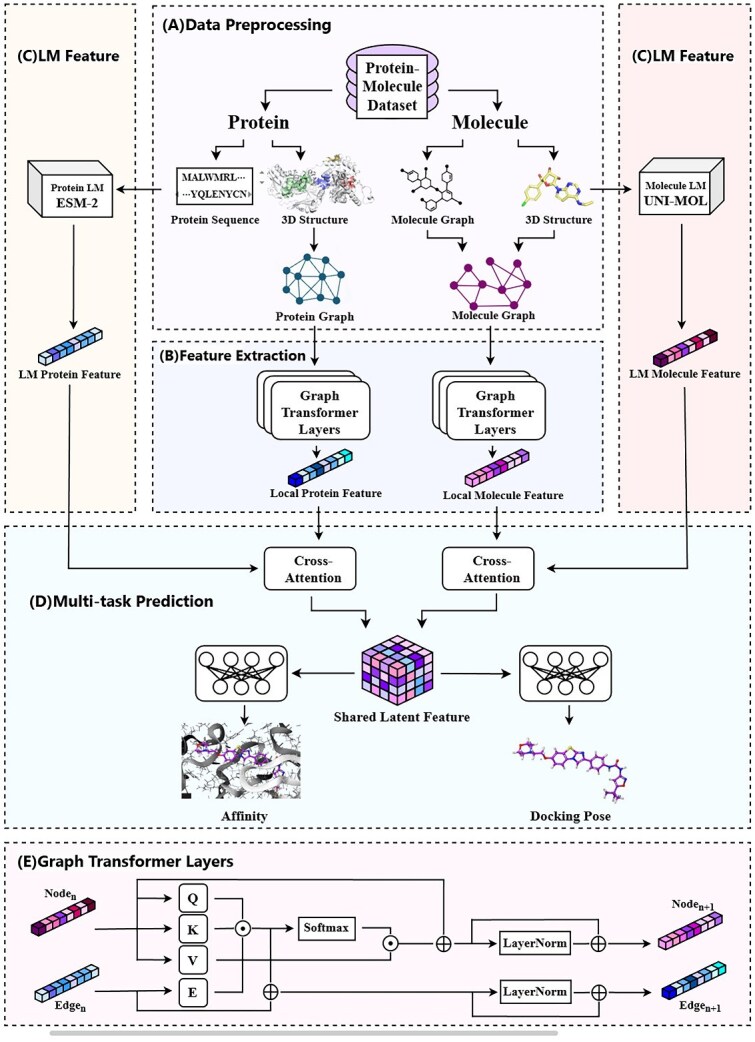
Overview of the model pipeline: (A) data preprocessing module, (B) feature extraction module, (C) LM feature module, (D) multi-task prediction module, and (E) graph transformer layers.

The objective of this study is to develop DTAP, a unified framework for the precise, simultaneous prediction of drug–target binding affinity and binding pose. This framework is designed to overcome several key limitations of previous models, including their single-task focus, poor generalizability to novel data, and frequent neglect of crucial 3D structural information. DTAP enhances its practical utility by facilitating sophisticated information exchange at multiple scales. It incorporates both local 3D structural information and powerful representations from pretrained LMs to learn from extensive unlabeled data, which significantly boosts its generalizability and ensures robust performance on unseen molecules. Furthermore, our use of a multi-task learning approach, where the binding affinity and docking pose prediction tasks share a common latent vector, enables effective knowledge transfer and helps to balance performance between screening and docking. The model’s architecture consists of four primary components: (i) Data preprocessing: We begin by generating decoy molecule–protein complexes and converting their 3D structural data into a graph-structured format. (ii) Graph-Transformer feature extraction: Two separate Graph-Transformer networks process the molecular and protein graphs. Unlike traditional graph neural networks, these networks use attention mechanisms to dynamically weigh atomic dependencies, effectively capturing both local and global features from molecular topological graphs. (iii) Pretrained LM feature extraction: A dedicated module leverages pretrained language models to learn rich, contextual representations from vast amounts of unlabeled data, enhancing the model’s ability to handle novel compounds. (iv) Multi-task prediction: The binding affinity and docking pose prediction tasks share a common latent vector while using task-specific decoders. This design promotes knowledge transfer and ensures a balanced performance across both screening and docking tasks. This comprehensive workflow is further illustrated in the accompanying Fig. 2, demonstrating how DTAP integrates these components for practical and effective drug discovery.

### Data preprocessing

#### Drug representation

Most small molecule feature extraction methods based on the SMILES sequences of drugs struggle to capture structural information, particularly 3D conformational details, like DGDTA [8] and BiComp-DTA [9]. Therefore, in this study, we employ molecular graphs of drugs to extract features. The graph-structured data effectively captures atomic characteristics, spatial configurations, and bond relationships within molecules. Therefore, we represent molecules as $G_{M}=\left (N_{M},E_{M}\right )$, where $N_{M}\ $denotes node features and $E_{M}\ $represents edge features. For node features, we employ one-hot encoding to represent atomic types, valence states, and other atomic properties, while incorporating 3D coordinate information for each atom. Regarding edge features, we apply one-hot encoding to characterize bond types and their participation in ring structures. In the PDBbind dataset, we utilized the docked molecular PDB files and the RDKit toolkit to construct molecular models. Atomic 3D coordinates were extracted from the PDB files, while node and edge features for graph construction were derived from the molecular models. These elements were integrated to form structured graph data. For the Davis and KIBA datasets, molecular representations were generated using SMILES strings in conjunction with RDKit. Inspired by previous research, these features have been demonstrated to effectively describe molecular properties [29, 30]. The information is shown in Tables 2 and 3.

**Table 2:** Representation of node features and characteristics of molecules

**Node feature**	**Content**
Atom type	[ C, N, O, F, P, S, Cl, Br, I, B, Si, Fe, Zn, Cu, Mn, Mo, other ] (one-hot)
Atomic Position X	X-coordinate of the atom (Integer)
Atomic Position Y	Y-coordinate of the atom (Integer)
Atomic Position Z	Z-coordinate of the atom (Integer)
Degree	[ 0, 1, 2, 3, 4, 5, other ] (one-hot)
Formal charge	Electrical charge (Integer)
Radical electrons	Number of radical electrons (Integer)
Hybridization	[ sp, sp2, sp3, sp3d, sp3d2, other ] (one-hot)
Is aromatic	[ 0, 1 ] (one-hot)
Num of hydrogens	[ 0, 1, 2, 3, other ] (one-hot)
Chirality type	[ R, S, other ] (one-hot)

**Table 3:** Representation of edge features and characteristics of molecules

**Edge feature**	**Content**
Bond type	[ Single, Double, Triple, Aromatic ] (one-hot)
Is conjugated	[ 0, 1 ] (one-hot)
Is in ring	[ 0, 1 ] (one-hot)
Stereo	[ StereoNone, StereoAny, StereoZ, StereoE ] (one-hot)

#### Protein representation

(1) Protein sequence representation

A protein is a biological macromolecule composed of multiple amino acids connected by peptide bonds, with each letter in the protein sequence corresponding to an amino acid (for instance, “A” stands for alanine and “D” for aspartic acid). Protein sequence representation refers to the process of converting a protein’s amino acid chain into a numerical format that can be interpreted and analyzed computationally. Given that protein sequences vary in length, we assign a fixed initial dimension of N (Usually set as 1000) to each sequence. Sequences shorter than N amino acids are padded with zeros, whereas those longer than N are truncated. Subsequently, using a predefined character-to-number mapping (such as converting “A” to “1” and “D” to “2”), the sequence is transformed into an integer-encoded feature. This method effectively transforms symbolic biological sequences into a machine-readable vectorial form, while ensuring that each amino acid is uniquely and unambiguously encoded.

(2) Protein graph representation with 3D information

Certain approaches perform feature extraction based solely on sequence data, like DGDTA [8] and BiComp-DTA [9]. However, protein amino acid sequences alone are insufficient to comprehensively capture their intricate structural information. In this study, we integrate both sequential and structural information to perform feature extraction for proteins. The 3D structure of proteins plays a crucial role in molecular–protein interactions, prompting us to employ PDB files containing 3D structural information as input. For proteins in the Davis and KIBA datasets that lack experimentally determined 3D structures and are only represented by amino acid sequences, we employed AlphaFold2 to predict their 3D structures. These predicted structural models were subsequently incorporated as part of the training data [31]. AlphaFold2 demonstrates high accuracy in protein structure prediction. We represent the protein’s graph structure as $G_{P}=\left (N_{P},E_{P}\right )$, where $N_{P}$ denotes node features and $E_{P}$ represents edge features. For node features, we utilize one-hot encoding to capture residue types and incorporate additional structural information such as dihedral angles. The edge features include residue connectivity information and other relevant features. Previous studies have demonstrated that using a 10.0Å cutoff threshold for determining residue connectivity (where residues with minimal distances below 10.0Å are considered connected) yields satisfactory performance [17]. Therefore, we adopt this well-established hyperparameter in our approach. Furthermore, edge features encompass spatial relationships such as C$\alpha $-O distances. To maintain numerical stability, we apply empirical scaling to certain features to constrain their ranges and prevent excessively large or small values. These features have been empirically validated in prior research as effective descriptors of protein characteristics and structural properties [32]. The information is shown in Tables 4 and 5.

**Table 4:** Representation of node features and characteristics of proteins

**Node feature**	**Content**
Residue type	[ G, A, V, L, I, P, F, Y, W, S, T, C, M, N, Q, D, E, K, R, H, metal, other ] (one-hot)
Max distance	Max distance of all atoms in residue (Integer)
Min distance	Min distance of all atoms in residue (Integer)
Distance of C$\alpha $-O	Distance of C$\alpha $-O (Integer)
Distance of O-N	Distance of O-N (Integer)
Distance of N-C	Distance of N-C (Integer)
Phi ($\phi $)	C’-N-C$\alpha $-C’ dihedral angle (Integer)
Psi ($\psi $)	N-C$\alpha $-C’-N dihedral angle (Integer)
Omega ($\omega $)	C$\alpha $-C’-N-C$\alpha $ dihedral angle (Integer)
Chi1 ($ \chi $1)	N-C$\alpha $-C$\beta $-C$\gamma $ dihedral angle (Integer)

**Table 5:** Representation of edge features and characteristics of proteins

**Edge feature**	**Content**
Is connected	[ 0, 1 ] (one-hot)
C$\alpha $ distance	C$\alpha $-C$\alpha $ distance between two residues (Integer)
Centroid distance	Centroid distance between two residues (Integer)
Max distance	Max atomic distance between two residues (Integer)
Min distance	Min atomic distance between two residues (Integer)

### Graph-Transformer feature extraction module

Graph-Transformer is a deep learning model that integrates the strengths of GNNs and Transformer architectures, demonstrating superior performance in processing graph-structured data. Unlike traditional graph neural networks that rely on fixed neighbor aggregation rules, Graph Transformer dynamically assigns weights to neighboring nodes through an attention mechanism. It can flexibly model long-distance dependencies between atoms or residues, thereby learning potential long-range interactions, while simultaneously mining both local and global information in molecular or protein topological graphs. Within the Graph-Transformer feature extraction module of our model, the architecture bifurcates into two parallel branches designed to process graph-structured data of molecules and proteins, respectively. Each branch employs a dedicated Graph-Transformer network to iteratively update node features and edge features for both molecular and protein representations, as depicted in Fig. 2B. The detailed process of Graph Transformer is depicted as Fig. 2E. 


(1) \begin{align*} Q_{M} &= W_{Q} N_{M}, \end{align*}

(2) \begin{align*} K_{M} &= W_{K} N_{M}, \end{align*}

(3) \begin{align*} V_{M} &= W_{V} N_{M}, \end{align*}

(4) \begin{align*} E_{M} &= W_{E} E_{M}, \end{align*}

(5) \begin{align*} W_{M} &= \mathrm{softmax}\left( \frac{Q_{M} K_{M}^{T}}{\sqrt{d_{k}}} \cdot E_{M} \right), \end{align*}

(6) \begin{align*} \mathrm{Head}_{M} &= W_{M} \cdot V_{M}, \end{align*}

(7) \begin{align*} \mathrm{MultiHead}_{M}^{\mathrm{Node}} &= \mathrm{Concat}(\mathrm{Head}_{M_{1}}, \ldots, \mathrm{Head}_{M_{n}}) \cdot W^{O}, \end{align*}

(8) \begin{align*} \mathrm{MultiHead}_{M}^{\mathrm{Edge}} &= \mathrm{Concat}(W_{M_{1}}, \ldots, W_{M_{n}}) \cdot W^{E}. \end{align*}

Here, $N_{M}$ and $E_{M}$ denote the node features and edge features of the molecule, respectively. $W_{Q}, W_{K}, W_{V}, W_{E}, W^{O}$, and $W^{E}$ represent separate learnable parameter matrices. ${Head}_{M}$ represents the result of a single-node attention head, while MultiHead denotes the final outcome of the multi-head attention mechanism. 


(9) \begin{align*} N_{M}^{\prime} &= N_{M} + \mathrm{MultiHead}_{M}^{\mathrm{Node}}, \end{align*}

(10) \begin{align*} E_{M}^{\prime} &= E_{M} + \mathrm{MultiHead}_{M}^{\mathrm{Edge}}, \end{align*}

(11) \begin{align*} N_{M}^{\mathrm{new}} &= N_{M}^{\prime} + \mathrm{FFN}(\mathrm{Norm}(N_{M}^{\prime})), \end{align*}

(12) \begin{align*} E_{M}^{\mathrm{new}} &= E_{M}^{\prime} + \mathrm{FFN}(\mathrm{Norm}(E_{M}^{\prime})). \end{align*}

where $N_{M}^{\prime }$ and $E_{M}^{\prime }$ represent the node features and edge features after residual connection, respectively, while $N_{M}^{\mathrm{new}}$ and $E_{M}^{\mathrm{new}}$ denote the final results after one layer of graph transformer processing. ${\mathrm{FFN}}\left (\cdot \right )$ stands for Feedforward Neural Network that is composed of MLPs. For protein representation, we employ an analogous feature extraction paradigm, and obtain the updated node feature as $N_{P}^{\mathrm{new}}$ and edge feature as $E_{P}^{\mathrm{new}}$.

### Pretrained large model feature extraction module

#### Drug pretrained large model representation

UNI-MOL [33] is a universal molecular pretrained framework designed specifically for capturing spatial and structural information. Its core innovation lies in the enhancement of the conventional Transformer architecture through the incorporation of 3D positional encoding and SE(3)-equivariant coordinate heads, enabling precise modeling of molecular spatial information. The SE(3)-equivariant design ensures the model’s invariance to molecular rotations and translations, while the 3D positional encoding effectively captures spatial relationships between atoms, allowing UNI-MOL to simultaneously process both 2D molecular structures and 3D conformational data. The input to Uni-Mol consists of molecular SMILES that are first converted into 3D conformations using a conformation generation algorithm based on ETKGD and Merck Molecular Force Field optimization. For pretraining, the authors compiled a large-scale dataset containing $\sim $209 million 3D molecular conformations generated from 19 million unique molecules. Each molecule is represented by atomic types and corresponding 3D coordinates, forming the dual input channels of the Transformer model.

In the Uni-Mol architecture, atom-level features are initialized through embeddings of atomic types, while pair-level features are initialized by computing rotationally and translationally invariant Euclidean distances between atom pairs. These pairwise spatial relationships are further refined using Gaussian kernel encodings. The model employs a modified Transformer backbone that integrates bidirectional communication between atom and pair representations: atom-level attention incorporates pairwise spatial biases, while pair-level encodings are updated through query–key interactions between atoms. To effectively learn from unlabeled data, Uni-Mol applies two key self-supervised pretraining tasks: (i) 3D position recovery, where the model learns to restore original coordinates from perturbed inputs, and (ii) masked atom prediction, where the model predicts atom types with incomplete information. After a fix number of Transformer processing, Uni-Mol outputs fixed-length continuous embeddings. For each molecule, the final output is a 512-dimensional vector, either extracted from the classification token or obtained via mean pooling over atom embeddings. This output vector encodes comprehensive chemical, spatial, and relational features and serves as a general-purpose representation for downstream tasks. Experimental results demonstrate that UNI-MOL-derived molecular embeddings exhibit superior performance across multiple drug discovery tasks, including molecular property prediction, protein–ligand binding affinity estimation, and conformation generation. To enhance molecular feature representation, improve feature generalization capability, and better address the cold-start problem, we extracted molecule features with Uni-mol LM as $H_{U}$.

#### Protein pretrained large model representation

ESM-2 [34] stands as one of the most advanced protein language models currently available, with its core strength lying in extracting evolutionary information from billions of protein sequences through large-scale self-supervised learning. The model employs an Transformer architecture capable of capturing complex evolutionary patterns and structure–function relationships embedded within amino acid sequences. In terms of feature extraction, ESM-2 not only accurately characterizes the physicochemical properties of individual residues but also effectively encodes long-range interactions between residues. Empirical studies demonstrate that the feature representations generated by ESM-2 exhibit exceptional performance in various applications, including remote homology detection and enzyme activity prediction, while maintaining superior generalization capabilities even for low-sequence-similarity proteins. These attributes make ESM-2 particularly valuable for computational analysis of protein evolution and function prediction. In order to enhance the generalized representation of protein features and ensure that the model has a good prediction effect for novel proteins, we employes ESM-2 LM to extract protein features as $H_{E}$.

### Multi-task prediction

In the multi-task prediction module, firstly, we integrate features generated by the UNI-MOL pretrained molecular foundation model with molecular features updated by the Graph-Transformer network, thereby forming comprehensive molecular feature representations. 


(13) \begin{align*}& M = \mathrm{CrossAttention}(H_{U}, N_{M}^{\mathrm{new}}, N_{M}^{\mathrm{new}})\end{align*}

Here, $N_{M}^{\mathrm{new}}$ represents the node features updated by the molecular Graph-Transformer, while $H_{U}$ denotes the pretrained LM features generated by UNI-MOL. The complete architecture is illustrated in Fig. 2C.

In the protein section, we integrate features generated by the ESM-2 pretrained protein LM with protein features updated by the Graph-Transformer network. Subsequently, we similarly employ cross-attention to fuse the features generated by the ESM-2 pretrained protein LM with the protein features updated by the Graph-Transformer network, thereby forming the complete protein features. 


(14) \begin{align*}& P = \mathrm{CrossAttention}(H_{E}, N_{P}^{\mathrm{new}}, N_{P}^{\mathrm{new}})\end{align*}

Here, $N_{P}^{\mathrm{new}}$ denotes the node features updated by the protein’s Graph-Transformer, while $H_{E}$ represents the pretrained LM features generated by ESM-2. The complete architecture is illustrated in Fig. 2C.

Then, we integrate molecular features with protein features through feature fusion, subsequently employing the combined feature representation for predictive modeling. The prediction module in our model can also be divided into two parts: the binding affinity prediction task and the docking pose RMSD prediction task. We aim to make those two tasks share a common latent vector and task-specific decoders, enabling cross-task knowledge transfer. Therefore, we add a parameter sharing module in the prediction module and then use two MLPs to perform predictions simultaneously, as shown in Fig. 2D. 


(15) \begin{align*} A_{ij} &= \sigma\left( \left( W_{\mathrm{MA}} \cdot \mathrm{ReLU}(M_{i}) \right) \left( W_{\mathrm{PA}} \cdot \mathrm{ReLU}(P_{j}) \right)^{T} \right), \end{align*}

(16) \begin{align*} J_{ij} &= \tanh\left( M_{i} \cdot P_{j} \right), \end{align*}

(17) \begin{align*} X_{ij} &= \sum_{i=1}^{N_{M}} \sum_{j=1}^{N_{P}} \left( A_{ij} \cdot J_{ij} \right), \end{align*}

(18) \begin{align*} \hat{y}_{ij} &= \mathrm{MLP}_{\mathrm{affinity}}(X_{ij}), \end{align*}

(19) \begin{align*} \hat{z}_{ij} &= \mathrm{MLP}_{\mathrm{RMSD}}(X_{ij}), \end{align*}

(20) \begin{align*} \mathrm{Loss} &= \alpha_{1} \cdot \mathrm{MSE}(y_{ij}, \hat{y}_{ij}) + \alpha_{2} \cdot \mathrm{MSE}(z_{ij}, \hat{z}_{ij}) \end{align*}

Here, $M_{i}$ denotes the features of the $i$th molecule, $P_{j}$ represents the features of the $j$th protein, $W_{\mathrm{MA}}$ and $W_{\mathrm{PA}}$ are two learnable matrix parameters, and $A_{ij}$ is the weight matrix for molecular–protein binding features. $ReLU\left (\cdot \right )$, $tanh\left (\cdot \right )$, and $\sigma \left (\cdot \right )$ denote three distinct activation functions. Research has demonstrated that this weight matrix effectively captures the importance differences between various components of molecules and proteins. $J_{ij}$ corresponds to the molecular–protein binding features, and $A_{ij}$ is the latent feature shared by both tasks, while $N_{M}$ and $N_{P}$ indicate the numbers of molecules and proteins, respectively. ${\hat{y}}_{ij}$ and ${\hat{z}}_{ij}$ represent the final prediction results output by the MLPs. The objective function of the model is to minimize the $Loss\left (\cdot \right )$, where the ${\mathrm{MSE}}\left (\cdot \right )$ is defined as the average of the squared differences between the predicted values and the true values.$\alpha _{1}$ and $\alpha _{2}$ represent the weight of the task. We employed the GradNorm method from the LibMTL library to dynamically adjust task weights by computing the gradient strength of each task with respect to the shared parameters and incorporating the relative training progress of each task. This approach adaptively balances the contributions of different tasks and prevents any single task from dominating the training process [35].

## Results

### Experimental settings

In our experiment, DTAP was implemented using PyTorch. The Adam optimizer was employed for model training with a learning rate of 1e-4. Both molecular and protein embeddings were set to 128 dimensions, with a dropout rate of 0.2. The number of epochs was set to 1000, and early stopping was applied when necessary in some experiments. We utilized Nvidia RTX 3090 GPU and RTX A6000 for the experiments on various datasets. The specific experimental settings are provided in Table 6.

**Table 6:** Hyperparameter settings of DTAP

**Hyperparameter**	**Setting**
Number of graph transformer layers	10
Number of MLP layers	4
Batchsize	50
GPU memory usage (GB)	23.5
Training time (min/epoch)	20

### Evaluation metrics

We use Mean Squared Error (MSE), C-index (CI), and $r_{m}^{2}$ as evaluation metrics. MSE is the average of the squared differences between predicted values and true values, which can effectively measure the accuracy of model predictions. CI is used to measure the ranking consistency between the model’s predicted values and the true values. $r_{m}^{2}$ is a modified version of the conventional coefficient of determination ($R^{2}$), which serves to evaluate the goodness of fit. The detailed information for each evaluation criterion is as follows: 


(1) \begin{align*} \mathrm{MSE} &= \frac{1}{n}\sum_{i=1}^{n}\left(y_{i}-\hat{y}_{i}\right)^{2}, \end{align*}

(2) \begin{align*} \mathrm{CI} &= \frac{1}{Z}\sum_{i>j}\left[I\left(y_{i}>y_{j}\right)\cdot\left(I\left(\hat{y}_{i}>\hat{y}_{j}\right)\right.\right. \notag \\ &\quad \left.\left. + 0.5\cdot I\left(\hat{y}_{i}=\hat{y}_{j}\right)\right)\right], \end{align*}

(3) \begin{align*} r_{m}^{2} &= R^{2} \times \left(1-\sqrt{R^{2}-R_{0}^{2}}\right) \end{align*}

where $y_{i}$ represents the true value, ${\hat{y}}_{i}$ denotes the corresponding predicted value, and $I\left (\cdot \right )$ is the indicator function. $R^{2}$ is the coefficient of determination, and $R_{0}^{2}$ is the $R^{2}$ obtained from regression through the origin.

### Performance of DTAP on the drug–target affinity and docking pose prediction tasks

In this section, we evaluate the performance of DTAP across two critical tasks: DTA prediction and docking pose prediction. We first assess its DTA prediction capabilities on the Davis and KIBA datasets. We then evaluate its joint performance-specifically, its ability to simultaneously predict DTA and docking poses-using the PDBbind and CASF2016 dataset.

**Figure 3:**
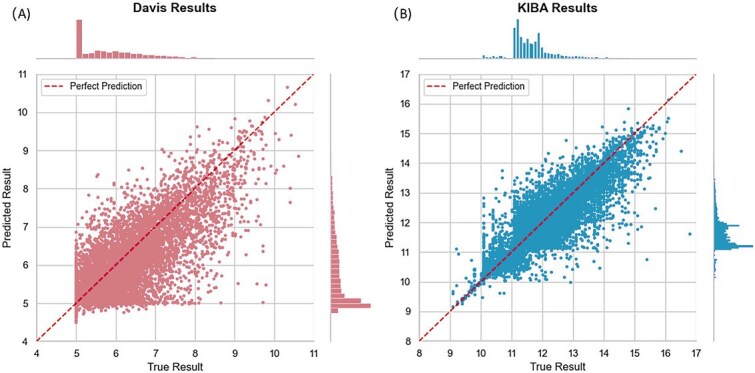
Correlation between predicted affinity values and true affinity values: (A) Davis dataset, and (B) KIBA dataset.

For Davis dataset, we selected 24 616 molecular–protein interaction affinity data points for model training, and employed five-fold cross-validation for performance evaluation. For performance comparison, we benchmarked against models tested on the same dataset in recent years, citing original paper results when our test data performance did not exceed reported values. Specific results are shown in Table 7, where the top-performing result is bolded and the second-best is underlined. Our model outperformed all others in MSE, CI and $r_{m}^{2}$ metrics, achieving a 0.03 improvement in MSE, a 0.005 improvement in CI and a 0.029 improvement in $r_{m}^{2}$ over the previous best models. The correlation plot between predicted and true affinity values (with negative logarithm scaled) in the Davis dataset is presented in Fig. 3A. As can be observed from the figure, both the predicted and true values are distributed $\sim $5 and are in close proximity to the prefect fitting line.

**Table 7:** Performance comparison of DTA prediction on the Davis dataset

**Model**	**MSE$\downarrow $**	**CI$\uparrow $**	**r2$\uparrow $**
DeepDTA (2018) [36]	0.261	0.878	0.650
GraphDTA (2021) [11]	0.229	0.893	0.692
TransVAE-DTA (2024) [37]	0.332	0.870	0.572
GDilatedDTA (2024) [38]	0.237	0.885	0.686
FL-DTA (2024) [39]	0.260	0.883	–
GramSeq-DTA (2025) [40]	0.261	0.796	–
OUR	**0.199**	**0.898**	**0.721**

For KIBA dataset, we selected 117 948 molecular–protein interaction affinity data points for model training, and employed five-fold cross-validation for performance evaluation, using the same evaluation metrics as in the Davis dataset. Specific results are shown in Table 8, where the top-performing result is bolded and the second-best is underlined. Our model outperformed all others in MSE and CI metrics, achieving a 0.015 improvement in MSE, a 0.004 improvement in CI over the previous best models. While our method achieves the same $r_{m}^{2}$ value as GDilatedDTA, it outperforms GDilatedDTA significantly in terms of the MSE and CI metrics. The correlation plot between predicted and true affinity values in the KIBA dataset is presented in Fig. 3B. As can be observed from the figure, both the predicted and true values are distributed around 11–12 and are in close proximity to the prefect fitting line.

**Table 8:** Performance comparison of DTA prediction on the KIBA dataset

**Model**	**MSE$\downarrow $**	**CI$\uparrow $**	**r2$\uparrow $**
DeepDTA (2018) [36]	0.194	0.863	0.691
GraphDTA (2021) [11]	0.203	0.888	0.760
TransVAE-DTA (2024) [37]	0.254	0.822	0.633
GDilatedDTA (2024) [38]	0.156	0.876	**0.775**
FL-DTA (2024) [39]	0.167	0.880	–
GramSeq-DTA (2025) [40]	0.355	0.832	–
OUR	**0.141**	**0.892**	**0.775**

**Figure 4:**
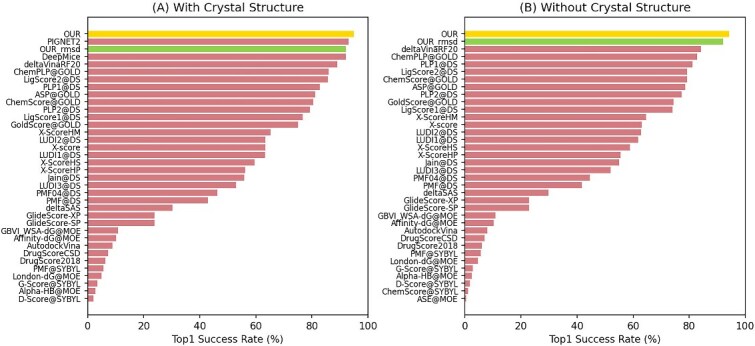
The Top1 success rate of pose RMSD prediction is shown, (A) results in the test set containing native structures, (B) results in the test set excluding native structures.

**Figure 5:**
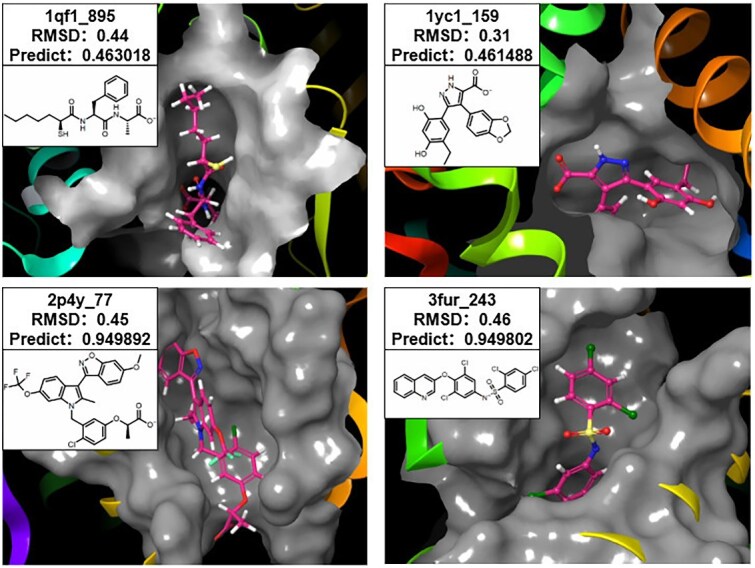
Some Top1 pose RMSD and predicted scores.

In the simultaneous prediction of affinities and RMSD values of docking poses, we used PDBbind for training and CASF2016 for testing. To test the performance of TDAP on predicting the RMSD values of docking pose, the Top1 success rate is used to evaluate the prediction results. The Top1 success rate represents the ratio that the highest-ranked docking pose is similar to the native pose (with RMSD $\leq $ 0.2 nm). For a comprehensive performance evaluation, we compare our results with the latest methods, PigNet2 (2024) and DeepMiCE (2025), and the baseline methods in CASF-2016. Specifically, PigNet2 constructs a protein–ligand interaction prediction model using a physics-informed graph neural network, whereas DeepMiCE builds a molecular docking framework based on a graph transformer network and a mixture density network. The Top1 success rate of our model’s structural RMSD prediction was 95.9$\%$ in the test set containing native poses and 95.2$\%$ in the test set without native poses. Meanwhile, our model achieved a Top1 success rate of 92.1$\%$ in structure RMSD prediction for individual tasks, and this metric reached 91.5$\%$ in a test set that does not include native conformations. The comparative results against other methods are presented in Fig. 4. The results of our model are highlighted for both multi-task and single task. It is obvious that the multi-task model has better performance than the single task on RMSD pose prediction . And our model achieved the highest Top1 success rate compared with all the other models, including the state-of-the-art models. The RMSD values and predicted scores for some Top1 poses are shown in Fig. 5. As demonstrated by the selected examples, DTAP provides accurate predictions of RMSD values.

**Figure 6:**
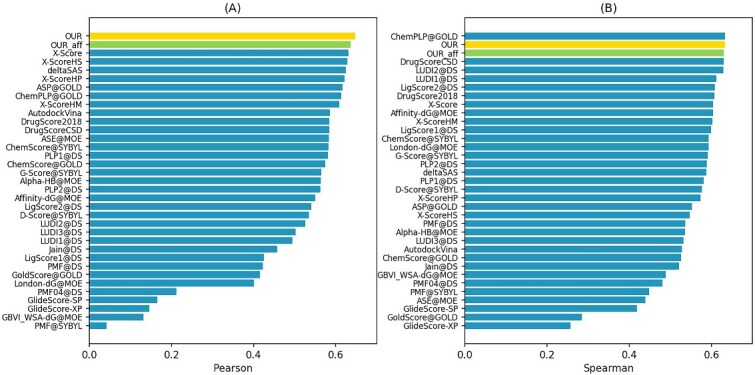
(A) PCC results of correlation between predicted and true affinity values, (B) SCC results of ranking ability.

At the same time, we used the Pearson correlation coefficient (PCC) to measure the correlation between the model’s predicted values and the labeled binding affinity, and the Spearman correlation coefficient (SCC) to measure the model’s ranking ability for different molecules of the same protein. The specific results compared with other baselines are shown in Fig. 6. The results of our model are highlighted. The bars labeled “OUR” represent the ability of DTA prediction with multi-task, while the bar labeled “OUR_aff” corresponds to the results of the single task of our model . It can be seen from the figure that our model performed best in PCC and ranked second in SCC, and the multi-task model DTAP have better performance than single tasks on DTA prediction.

### DTAP generalization performance

In the evaluation of the model’s generalization capability on cold-start datasets, we divided the testing data into three directions: molecular cold-start dataset (M-cold start), protein cold-start dataset (P-cold start), and molecule-protein cold-start dataset (M-P-cold start). For each category, we adopted a random splitting strategy. Specifically, in the molecule cold-start setting, the Davis dataset (containing 68 molecules, 361 proteins, and 24 616 molecule–protein pairs) was split by distinct molecules into subsets of 54 and 14 molecules, resulting in a training set (54 molecules, 361 proteins, and 19 548 pairs) and a test set (14 molecules, 361 proteins, and5068 pairs). In the protein cold-start setting, a similar approach was applied but based on distinct proteins. For the protein–molecule cold-start dataset, the same principle was followed, but splitting was performed to ensure that both molecules and proteins in the test set were distinct from those in the training set (while this may lead to the loss of some data, it guarantees that no test molecule or protein appears in the training set). After splitting, in the molecular cold-start dataset, each drug present in the training set is absent from the test set. In the protein cold-start dataset, each protein present in the training set is absent from the test set. In the molecule–protein cold-start dataset, both every drug and every protein present in the training set are absent from the test set. We constructed these cold-start datasets based on the Davis dataset for training and testing. Detailed dataset information is provided in Table 9.

**Table 9:** Cold-start dataset information, M-cold (each molecule that appears in the training set does not appear in the test set), P-cold (each protein that appears in the training set does not appear in the test set), M-P-cold (both molecule and protein which appear in the training set do not appear in the test set).

**Dataset**	**M (Train)**	**P (Train)**	**C (Train)**	**M (Test)**	**P (Test)**	**C (Test)**
M-cold start	54	361	19 548	14	361	5068
P-cold start	68	289	19 720	68	72	4896
M-P-cold start	54	289	15 660	14	72	1008

M represents molecules, P represents proteins, and C represents complexes

**Figure 7:**
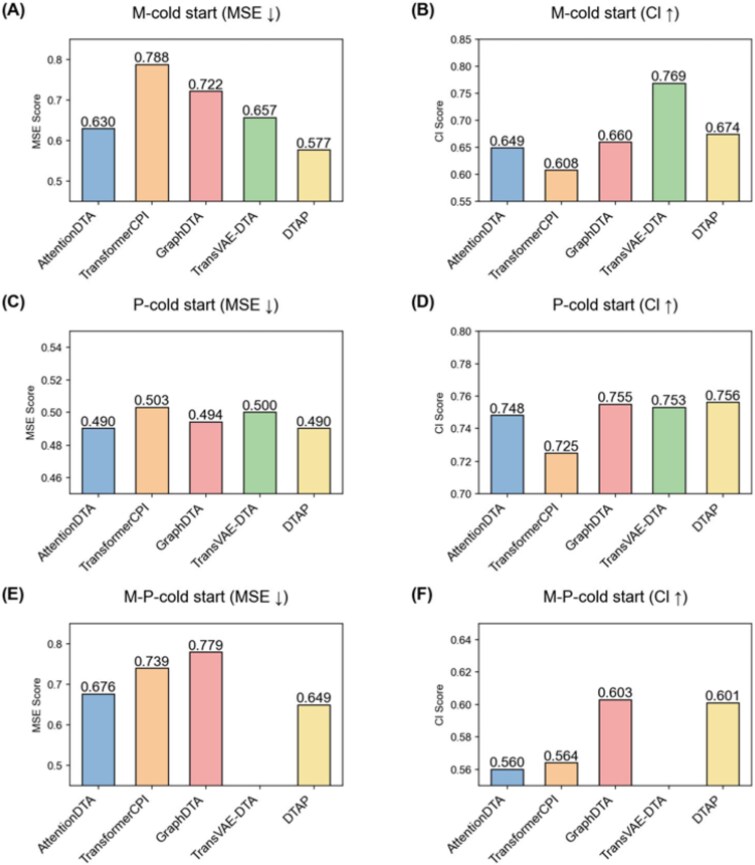
The comparison with other methods on cold-start datasets. (A) MSE comparison on Molecule-cold start datasets, (B) CI comparison on Molecule-cold start datasets, (C) MSE comparison on Protein-cold start datasets, (D) CI comparison on Protein-cold start datasets, (E) MSE comparison on Molecule-Protein-cold start datasets, (F) CI comparison on Molecule-Protein-cold start datasets.

We use MSE and CI as validation metrics, and select models tested in the same dataset in recent years for performance comparison. If the test data performance is not better than the original paper data, we cite the original paper data for display. The specific results are shown in Fig. 7. Our model performs better than other models in the MSE metric in the molecular cold-start dataset, improves MSE by 0.053 compared with the second-ranked model, and ranks second in the CI metric. In the protein cold-start dataset, our model ranks first in the MSE metric and in the CI metric. In the molecule–protein cold-start dataset, our model performs better than other models in the MSE metric and ranks second in the CI metric. This shows that the inclusion of pretrained LM features significantly improves the generalization ability of our model.

### Ablation experiments

To test the improvement of pretrained LM features and the feature fusion methods on our model’s prediction capability and generalization ability, we conducted ablation experiments, mainly focusing on the impact of adding UNI-MOL and ESM-2 LMs. The specific results are shown in Table 10, where bold values indicate the final results of the complete model. As shown in the table, incorporating both UNI-MOL and ESM-2 features improves the model’s predictive accuracy, and fusing these features via cross-attention yields superior performance over direct concatenation. The model with UNI-MOL and ESM-2 features achieves a 0.027 reduction in MSE compared with the baseline model without these features.

**Table 10:** Ablation study

**Model**	**MSE$\downarrow $**	**CI$\uparrow $**
DTAP (Without LLM)	0.223	0.876
DTAP (Without UNI-MOL)	0.211	0.883
DTAP (Without ESM-2)	0.214	0.882
DTAP (Without cross-attention)	0.215	0.879
DTAP	**0.196**	**0.897**

### DTAP interpretability performance

**Figure 8:**
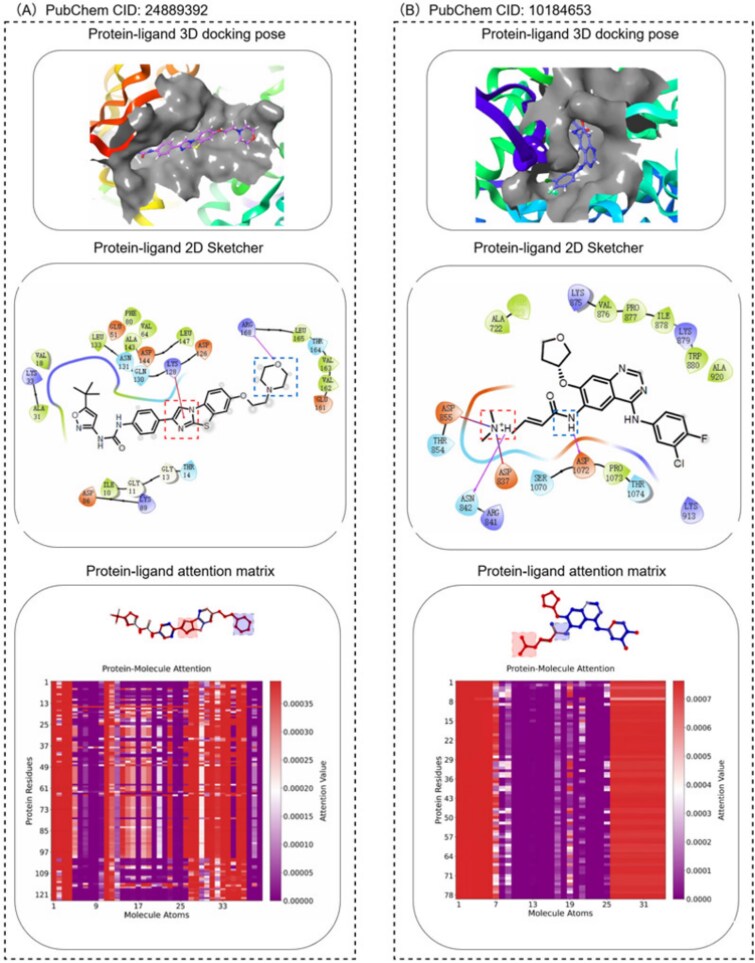
Interpretability of the model attention mechanism visualized through 3D docking, 2D diagrams, and attention heatmaps for two protein–ligand complexes. (A) An Exemplary Case: PubChem CID:24889392. (B) An Exemplary Case: PubChem CID:10184653.

In our model, after the fusion of protein features and molecular features, we used an attention module to obtain a weight matrix with the number of rows equal to the number of molecular atoms and the number of columns equal to the number of protein residues, to analyze whether the model effectively highlights the important parts in molecular–protein docking. The specific effects are shown in Fig. 8. The protein–ligand 3D docking pose was obtained from the Schrödinger software, while the Protein–ligand 2D Sketcher was generated by the same software to represent the docking results in a 2D format. The protein–ligand attention matrix visualizes the attention weights from our model, with the x-axis representing ligand atoms and the y-axis representing protein amino acid residues. The color intensity of each cell corresponds to the attention weight magnitude, as shown in the color bar on the right. Additionally, the ligand atoms are colored based on their aggregated attention weights. Colored rectangles are overlaid on regions with high attention values to emphasize key interaction sites.

As shown in Fig. 8, the regions identified as critical by the docking software align closely with the high-attention regions highlighted by our model. This strong correspondence demonstrates that the model successfully learns to assign greater importance to specific structural locations. These locations are also shown in the docking results to be in close proximity, which is a key factor determining whether molecular interactions occur.

## Discussion

In this study, we propose an innovative model for predicting both drug–target binding affinity and docking pose that employs Graph-Transformer networks to extract features of molecules and proteins, incorporates pretrained LMs to enhance generalization capability, and simultaneously predicts interaction strength from both energetic and structural perspectives. Experimental results demonstrate that our model achieves strong performance in both drug–target binding affinity prediction and docking pose prediction. Its excellent performance on cold-start datasets indicates robust predictive accuracy when encountering novel molecules or proteins. Interpretability analyses confirm that our model correctly identifies and assigns higher weights to key interaction sites, which have been validated in actual docking studies. Despite these achievements, our model has limitations. First, although we utilize partial features and incorporate pretrained large-model features, our integration of multimodal information remains insufficient. For instance, we do not include molecular 1D sequence information. Second, our protein features are derived from entire protein structures, while the truly critical information lies in binding pockets. This approach consumes more computational resources without commensurate performance gains. Finally, our current model does not possess the capability to generate molecular conformations, which represents a direction for our future research. In subsequent work, we plan to integrate diverse sources of information and explore additional predictive dimensions to enhance both accuracy and interpretability. We also intend to incorporate rotation and translation invariance modules to improve the model’s generalizability and evaluate its performance on more diverse datasets. These efforts are expected to contribute to the advancement of small-molecule drug screening research.

**Key Points**

- We developed DTAP, a unified multi-task learning framework that simultaneously predicts drug–target docking pose quality and binding affinity.
- To enhance generalizability to novel molecules and proteins, DTAP fuses pretrained large model representations with intrinsic molecular/protein representations, leveraging pretrained models’ ability to learn contextual features from unlabeled data.
- DTAP employs multi-modal representations encompassing both sequence data and 3D structural details of proteins and molecules, thereby capturing critical spatial interactions essential for accurate drug–target interaction prediction.
- Validation of DTAP on the Davis, KIBA, CASF2016, and PDBbind datasets demonstrates its robust performance in predicting both drug–target affinity and the root-mean-square deviation between docking poses and native structures.

## Data Availability

The datasets used in this study are all publicly available. The source code and data for DTAP can be downloaded from GitHub (https://github.com/OvO15527/DTAP).
